# Preparation and Physicochemical Properties of Blend Films of Feather Keratin and Poly(vinyl alcohol) Compatibilized by Tris(hydroxymethyl)aminomethane

**DOI:** 10.3390/polym10101054

**Published:** 2018-09-20

**Authors:** Xunjun Chen, Shufang Wu, Minghao Yi, Jianfang Ge, Guoqiang Yin, Xinming Li

**Affiliations:** 1 Green Chemical Engineering Institute, Zhongkai University of Agriculture and Engineering, Guangzhou 510225, China; SFWu2018@163.com (S.W.); MHYi0848@163.com (M.Y.); ge650704@163.com (J.G.); yingq007@163.com (G.Y.); lixinming@sina.com (X.L.); 2 Guangzhou Key Laboratory for Efficient Utilization of Agricultural Chemicals, Guangzhou 510225, China

**Keywords:** blend film, feather keratin, poly(vinyl alcohol), tris(hydroxymethyl)aminomethane, solution-casing, physicochemical properties

## Abstract

Blend films of feather keratin (FK) and synthetic poly(vinyl alcohol) (PVA) that were compatibilized by tris(hydroxymethyl)aminomethane (Tris) were successfully prepared by a solution-casting method. The scanning electron microscopy (SEM) results showed that a phase separation occurred in the FK/PVA/Tris blended system. Analysis by Fourier transform infrared spectroscopy indicated that the main interactions between the three components were hydrogen bonds. In addition, X-ray diffraction analysis showed that the FK/PVA/Tris blend films were partially crystalline. The barrier properties, mechanical properties, and contact angles of the FK/PVA/Tris films were investigated to determine the effects of the PVA and Tris concentrations. More specifically, upon increasing the PVA content, the elongation at break, the hydrophilicity, and the oxygen barrier properties were enhanced. However, at a constant PVA content, an increase in the Tris content caused the oxygen permeability and the contact angle to decrease, while the tensile strength, elongation at break, and oxygen barrier properties were enhanced. These results indicated that the mechanical properties and gas resistance of the FK/PVA/Tris blend films could be successfully improved using the method described herein, confirming that this route provided a convenient and promising means to prepare FK plastics for practical applications.

## 1. Introduction

Due to their excellent mechanical properties and stable physicochemical properties, synthetic polymer materials are widely used in packaging. However, synthetic polymers are generally not biodegradable, and their use can result in environmental pollution. To address these issues, their replacement with biomaterials has received increasing attention, with examples including proteins [1], carbohydrates [2,3,4,5], and lipids [6]. Keratin, a protein found in abundance (80–90% protein content) in animal hair, feathers, and hooves, has received significant research interest. It is estimated that 3–4 billion pounds of feathers are generated annually as a byproduct of the poultry industry in the United States [7], in addition to >1.5 billion pounds in China [8]. Although feather keratin is easily available, biodegradable, and exhibits high tensile strength, its application in packaging is challenging due to its brittle nature. As such, the modification of keratin to meet packaging requirements through combination with other polymer materials and blend modification is necessary.

Poly(vinyl alcohol) (PVA) is a widely used, nontoxic, water-soluble, and biodegradable polymer material with good mechanical properties. More specifically, its tensile strength can reach 34.3 MPa, while its elongation at break can reach 450%. In addition, it exhibits excellent chemical stability, in addition to gas barrier, anti-fragrance, and film-forming performances. The use of PVA in combination with biomaterials such as chitosan [2,9,10,11], starch [3,4,12,13,14], soy protein isolate (SPI) [15,16], and silk protein [17] has therefore been reported. However, to the best of our knowledge, there have been few reports on the blend modification of PVA and feather keratin (FK) [18,19,20]. Specifically, Yao et al. [18,19] reported the extraction of FK by reduction of the preparation of dialdehyde starch cross-linked FK-based films blended with PVA by solution-casting techniques and found PVA incorporation could improve the mechanical properties, stability and water-resistance of keratin films; Ming et al. [20] successfully fabricated and characterized FK/PVA composite nanofibers through an electrospinning process. Thus, we herein report the use of PVA to improve the film-forming performance and mechanical properties of FK, and ultimately improve its suitability for use in the packaging field.

Moreover, we examine the application of a protein plasticizer, namely tris(hydroxymethyl)aminomethane (Tris), which has a molecular structure similar to that of glycerol (i.e., both contain three hydroxyl groups). Tris is also a weak organic base, and an aqueous solution of Tris has an approximate pH of 10.5. The dissolution of keratin using a Tris solution can therefore address the issue of protein denaturation upon dissolution in a strong inorganic alkali. Interestingly, there have been no reports on the use of Tris in FK/PVA composites. As such, we report the extraction of FK by partial oxidation with peracetic acid, followed by the preparation of Tris-plasticized FK-based films blended with PVA by solution-casting techniques. Ultimately, we wish to investigate the influence of the Tris plasticizer and PVA on the properties of cast FK keratin films.

## 2. Materials and Methods

### 2.1. Materials

The chicken feathers employed herein were collected from farmers’ markets in Guangzhou city (China). PVA (degree of polymerization = 1700, degree of alcoholysis = 99) was purchased from Aladdin Ltd. (Shanghai, China). Tris was obtained from the Shanghai Ebene Chemical Reagent Co., Ltd. (Shanghai, China), and peracetic acid was purchased from Guangzhou Chemical Ltd. (Guangzhou, China). All commercial reagents were of analytical grade and were used without further purification. Deionized water was employed as a solvent.

### 2.2. Extraction of Feather Keratin (FK)

FK was extracted according to the procedure reported by Ming et al. with slight modifications [21]. The cleaned chicken feathers were added to a 16 wt% solution of freshly prepared peracetic acid (1:12, *w*/*w*), and extraction was performed at 80 °C for 60 min under constant stirring. The extraction solution was then subjected to centrifugation at 4000 rpm for 5 min, and the centrifugal solution was passed through nylon mesh (75 mm). The obtained filtrate was subjected to dialysis (Oso-T8280 dialysis tubes, 8000–14,000, Union Carbide, Houston, TX, USA) against distilled water at 25 °C over 1 w, during which time the water was changed at 12 h intervals. The desired FK was precipitated upon dialysis. Following filtration, washing, and freeze-drying, the obtained FK was ground into 200-mesh and stored in a sealed bag prior to use.

### 2.3. Preparation of the FK/PVA/Tris Films

A Tris solution was prepared by adding Tris powder (6 g) to deionized water (94 g) at room temperature with continuous stirring over 5 min. A solution of FK was prepared by the addition of FK powder to the Tris solution with continuous stirring at 40 °C for 30 min. Mixtures containing various weight ratios of the 6% aqueous PVA solution were prepared as film-forming solutions, which were poured onto polypropylene dishes to prepare the FK/PVA/Tris blend films. Notably, the total solid content of FK and PVA (0.7 g) in the film-forming solution remains unchanged. These dishes were then dried in a humidity chamber at 25 °C and 50% relative humidity for 24 h, after which the films were easily removed from the dish by peeling. A series of FK/PVA/Tris films were coded as P-m-n, where m is the weight percentage of PVA relative to the total weight of FK and PVA in the film, and where n is the weight percentage of Tris relative to the total weight of FK and PVA in the film. For example, P-40-25 refers to an FK to PVA weight ratio of 60:40, and a Tris content of 25 wt% relative to the total weight of FK and PVA. It should be noted that as we wished to investigate the modification of these FK-based films, the maximum amount of incorporated PVA was 50% (P-50-30). The resulting blended films were conditioned at 25 °C and 50% relative humidity for 48 h prior to testing.

### 2.4. Preparation of the Control Films

A solution of FK was prepared by the addition of FK powder to the deionized water. The FK solution was adjusted to pH 8.5 with 0.1 mol/L NaOH solution, with continuous stirring at 40 °C for 30 min. Mixtures containing 40% weight ratios of the 6% aqueous PVA solution and various weight ratios of glycerol (0% and 15%) were prepared as film-forming solutions. The pH of the film-forming solution was again adjusted to pH 8.5 with 0.1 mol/L NaOH solution. The next steps are the same as the FK/PVA/Tris blend film preparation. The control films were coded as G-x-y, where x is the weight percentage of PVA relative to the total weight of FK and PVA in the film, and where y is the weight percentage of glycerin relative to the total weight of FK and PVA in the film.

### 2.5. Characterization

The surface morphologies of the film samples were observed by scanning electron microscopy (SEM, EVO 18; Carl Zeiss, Jena, Germany) at 15 kV accelerating voltage.

Fourier transform infrared spectroscopy (FTIR) was carried out for the film samples (Spectrum 100 infrared spectrometer, Perkin-Elmer, Fremont, CA, USA) using the attenuated total reflectance mode (ATR) between 4000 and 650 cm^−1^ (8 scans per wavenumber). Tris and the FK powder were tested using potassium bromide tableting between 4000 and 650 cm^−1^ (4 scans per wavenumber). The obtained spectra were analyzed using Omnic software (OMNIC 8.2).

The crystallinity of the sample was determined by powder X-ray diffraction (XRD, PW3040/60, PANalytical Company Ltd., Almoro, the Netherlands) with Cu Kα irradiation at an applied voltage of 40 kV (40 mA), a 2θ scan range of 5–50°, and a scan speed of 10°/min.

The tensile properties of the film samples were measured on a microcomputer-controlled electronic universal testing machine (CMT6503, Shenzhen MTS Test Machine Company Ltd., Shenzhen, China), according to the ASTM D 882 standard, with a speed of 10 mm/min and a fixture distance of 40 mm. The films were cut into samples measuring 75 mm × 10 mm, and the sample thicknesses were measured using a digital external micrometer (accurate to 0.001 mm). The measurements were conducted in triplicate and average values calculated.

Contact angle measurements were performed using an automatic contact angle meter (Theta, Biolin Scientific Ltd., Espoo, Finland). For this purpose, the film was placed flat on a glass slide and droplets of deionized water were extruded from the needle tube onto the film surface. The contact angles between the film samples and the water droplets were then measured. To compensate for departure of the film from the horizontal during preparation, the contact angle was evaluated as the average value of measurements made at the both sides of water drops.

The water vapor permeability (WVP) values of the films were measured using a water vapor transmittance tester (W3/030, Labthink Ltd., Jinan, China) at 38 °C with a gradient of 90–0% relative humidity across the film. All the film samples were cut into circles with diameters of 3 cm. The measurements were carried out in triplicate and average values calculated.

Finally, the oxygen permeability (OP) values of the films were measured using an oxygen permeability tester (VAC-VBS, Labthink Ltd., Jinan, China) according to the GB/T 1038-2000 standard with a test gas pressure of 1.01 × 10^5^ Pa and upper and lower degassing times of 4 h. All the film samples were cut into circles with diameters of 5.5 cm. The measurements were carried out in triplicate and average values calculated.

## 3. Results and Discussion

### 3.1. Morphology of the Films

All films were macroscopically uniform, yellowish and optically transparent films. The surfaces of the FK/PVA/Tris blend films look very smooth, which are smoother than the surfaces of the FK/PVA blend films [18,19], without visible cracks or holes. The appearance of these films did not change when different Tris and PVA ratios were used. All films could be easily peeled from the dish, except P-0-30. However, the appearance of the two sides of the film was different. The film side facing the casting plate was shiny, while the other side was dull, probably due to the polymer arrangement during solvent evaporation. This phenomenon was also observed for FK/sodium alginate blend films by Ming et al. [21].

**Figure 1:**
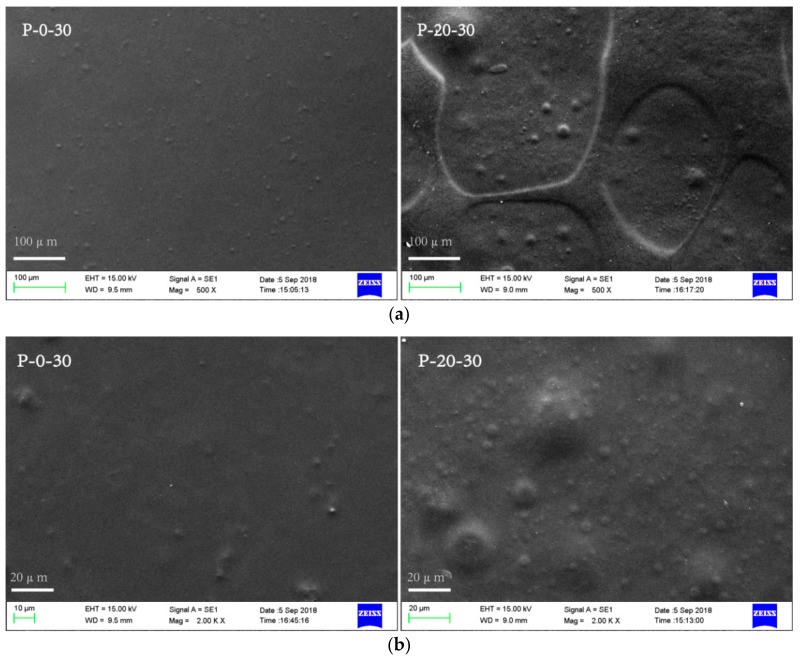

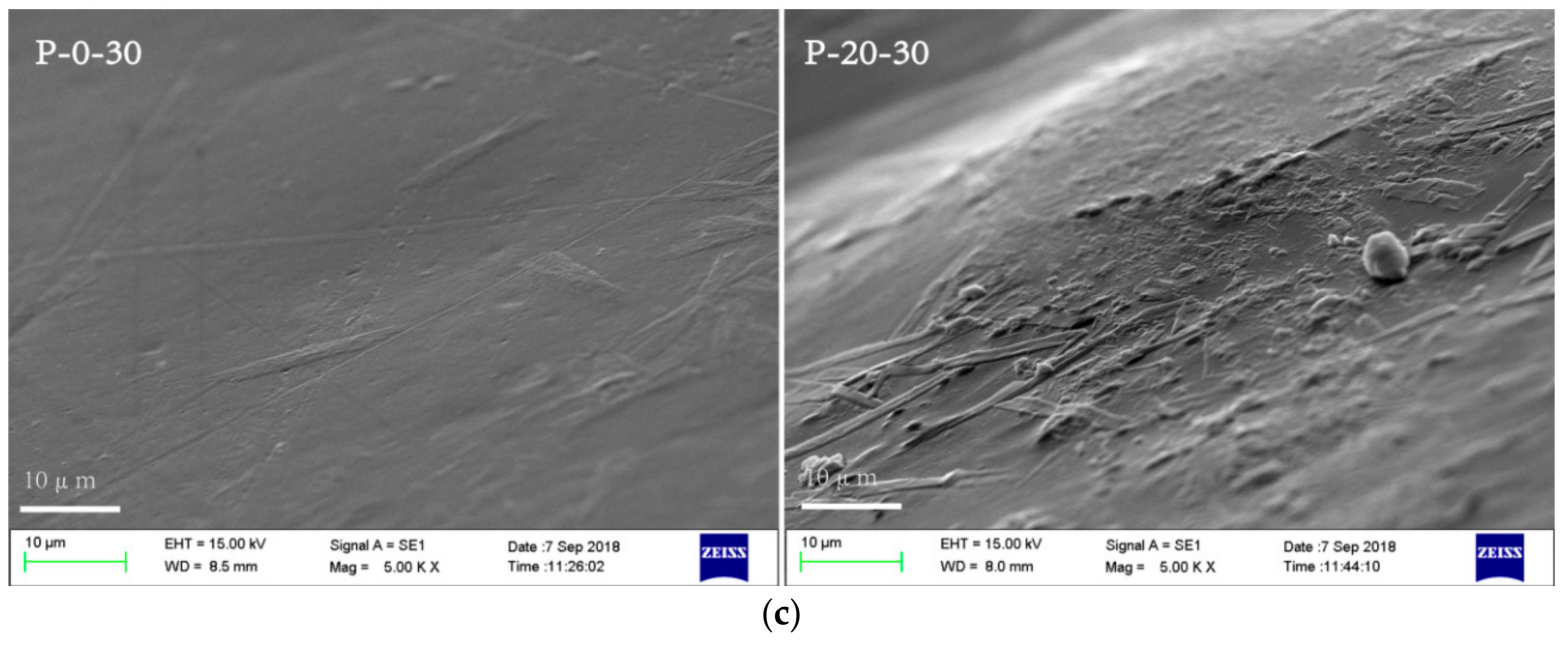
Representative scanning electron microscopy (SEM) images revealing the surface morphology (**a**) 500×, (**b**) 2000× and (**c**) the fracture morphology (5000×) of the feather keratin (FK)/poly(vinyl alcohol) (PVA)/tris(hydroxymethyl)aminomethane (Tris) blend films.

Scanning electron microscopy (SEM) observations were carried out to get a better insight of the homogeneity and the microstructure of the blend films. SEM images of the surface of the selected blend films (P-0-30 and P-20-30) are shown in Figure 1. It can be seen that the surface of P-20-30 was rougher than P-0-30, with more teardrop-shaped bulges revealed at higher magnifications. That is to say, as the PVA content increases, the surface of the blended membrane is less smooth, which can probably be attributed to the different coacervation kinetics of FK compared to PVA. The homogeneity of the blend films was more accurately judged by observing the fracture morphology of the blended films. P-0-30 exhibited a relatively smooth and homogeneous fracture surface. A rough fracture surface with a few small particles inside the matrix was observed for P-20-30, suggesting a phase separation caused by PVA aggregates in the FK/Tris matrix. In conclusion, the SEM results show that a phase separation occurred in the FK/PVA/Tris blended system. The phase separation phenomenon of the blend films becomes more serious with increasing PVA content.

### 3.2. FTIR Analysis

**Figure 2:**
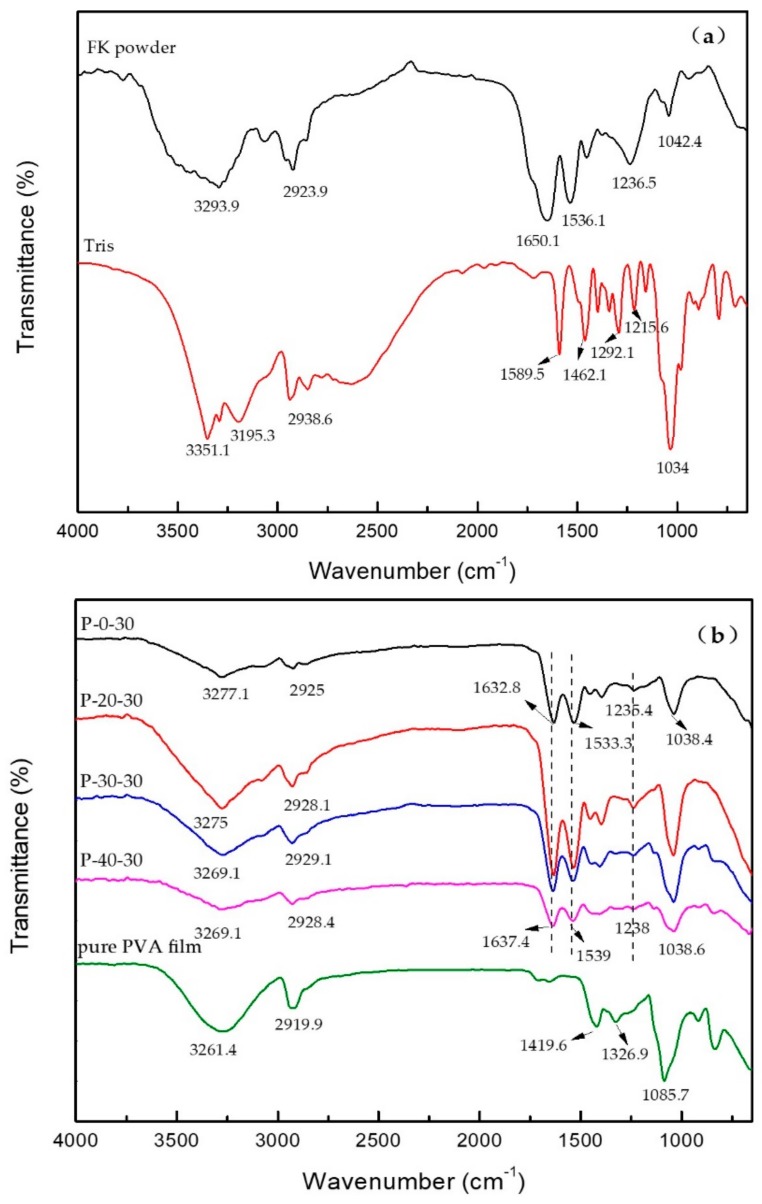
FTIR spectra of (**a**) the FK powder and Tris, and (**b**) the FK/PVA/Tris blend films and the pure PVA film.

Figure 2a shows the FTIR spectra recorded for the FK powder and the Tris. In the case of the FK powder, the signals observed at 3293.9, 2923.9, and 1650.1 cm^−1^ correspond to the O–H and N–H association peaks, the –CH stretching vibration peak, and the peak characteristic to the FK amide I band, respectively. Furthermore, the peaks at 1536.1, 1236.5, and 1042.4 cm^−1^ correspond to the characteristic absorption peak of the FK amide II band (i.e., the N–H bending vibration) [22], the FK amide III band (i.e., the C=O bending and C–N stretching vibrations) [23], and the C–O stretching vibration peak of FK, respectively. Moreover, in the FTIR spectrum of the Tris, the signals at 3351.1, 3195.3, 2938.6, 1589.5, 1462.1, 1292.1, 1215.6, and 1034 cm^−1^ correspond to the O–H stretching vibration, the N–H stretching vibration, the –CH stretching vibration, the N–H and C–H bending vibrations, and the C–N, the C–C, and the C–O stretching vibrations, respectively.

As expected, upon blending FK and Tris to form a film, significant changes were observed in the FTIR spectrum. More specifically, the characteristic absorption peaks of FK at 3293.9, 1650.1, 1536.1, and 1042.4 cm^−1^ were shifted to P-0-30 at 3277.1, 1632.8, 1533.3, and 1038.4 cm^−1^ (corresponding to the O–H and N–H association peaks, the amide I, II, and the C–O stretching vibrations, respectively), and the characteristic absorption peaks of Tris at 1589.5 cm^−1^ disappeared, which indicates the presence of hydrogen bonding between the Tris and FK molecules.

Figure 2b shows the FTIR spectra recorded for the pure PVA film and the FK/PVA/Tris blend films. In the case of the PVA film, the signals at 3261.4, 2919.9, 1419.6, 1326.9, and 1085.7 cm^−1^ correspond to the O–H stretching vibration, the –CH stretching vibration, the O–H and C–H bending vibrations, the CH–OH bending vibration [20], and the C–O stretching vibration, respectively.

As expected, upon blending FK, PVA and Tris to form a film, significant changes were observed in the FTIR spectrum. More specifically, the characteristic absorption peaks of FK/Tris at 1632.8, 1533.3, and 1235.4 cm^−1^ were shifted to 1637.4, 1539, and 1238, cm^−1^ (corresponding to the amide I, II, and III stretching vibrations, respectively). This could be attributed to the interactions taking place between the three pure components upon blending. Similarly, the N–H and O–H association peak of FK/Tris observed at 3277.1 cm^−1^ shifted to 3269.1 cm^−1^ for the blended membrane, which indicates the presence of hydrogen bonding between the Tris, FK and PVA molecules, confirming the compatibility between the three pure components [24].

It should be noted here that FTIR is a highly sensitive technique suitable for investigating protein conformations, where conformational changes can be determined through observation of the 1600–1700 cm^−1^ amido I region. More specifically, the bands located at 1610–1640 cm^−1^ can be assigned to β-sheets, those at 1640–1650 cm^−1^ correspond to random coils, the bands at 1650–1660 cm^−1^ correspond to α-helices, and those at 1660–1700 cm^−1^ correspond to β-turns [25,26]. The secondary structures of the samples were then analyzed using the Omnic software, as outlined in Table 1. Upon increasing the PVA content, the α-helix content increased in the FK secondary structure of the composite membrane, indicating that the stability of the composite membrane was improved [25]. Similarly, the β-sheet content was reduced, indicating an improvement in membrane toughness [27], while both the β-turn and random coil content were increased.

### 3.3. XRD Analysis

**Figure 3:**
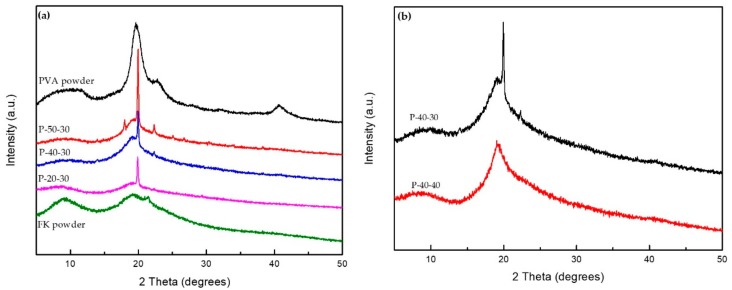
XRD patterns of: (**a**) the pristine PVA powder, P-50-30, P-40-30, P-20-30, and the pristine FK powder, (**b**) P-40-30 and P-40-40.

The XRD patterns of the pristine FK, pristine PVA, and FK/PVA/Tris blend films at 2θ = 5–50° are shown in Figure 3. Pristine PVA exhibited a broad diffraction peak at 19.5°, which was attributed to its 101 crystal reflection planes [28,29]. A weak diffraction peak was also observed at 40.7°. In addition, pristine FK gave broad diffraction peaks at 9.5° and 19.2°, which are typical fingerprints of the partially crystalline materials. Where no strong interactions are present between PVA, Tris and FK, the composite film will exhibit diffraction peaks corresponding to the pure component. However, for the prepared FK/PVA/Tris composite membrane, the two diffraction peaks attributed to FK (2θ = 9.5°) and PVA (2θ = 40.7°) disappeared and a new signal appeared at 19.9°. This indicates that a strong hydrogen bond is formed between the FK, PVA and Tris molecules, which destroys the regularity of the original molecules and affects the crystal structures of both FK and PVA. This was also confirmed by the change in the secondary structure suggested by FTIR analysis.

Furthermore, the XRD patterns of the FK/PVA/Tris blended films exhibited less intense signals at 9° and more intense signals at 19.9° than the pristine FK. These results indicate that the peak intensity at 19.9° increases with increasing PVA content, likely due to the high crystallinity of PVA; therefore, the FK/PVA/Tris blend films could be considered partially crystalline materials. Figure 3b shows that the intensity of the characteristic peak at 19.9° slightly decreased with increasing Tris content, indicating that the plasticizing induced by the addition of Tris reduces the relative crystallinity of the FK/PV/Tris blend to some extent.

### 3.4. Mechanical Properties

The effects of the PVA and Tris contents on the mechanical properties of the blend films are outlined in Table 2. From these results, it can be deduced that at equal Tris contents, the amount of PVA in the blend film directly influenced the tensile mechanical properties of the protein film. Specifically, with increasing PVA content, the elastic modulus decreased, reducing from 493.66 to 214.55 MPa upon moving from P-10-30 to P-50-30. In addition, the elongation at break increased from 1.75% to 179.83% on moving from P-10-30 to P-50-30. This result indicated that the addition of PVA addressed the issues relating to brittleness and the poor mechanical properties of the protein membrane. A similar result was reported when glycerol was employed for the plasticization of SPI/PVA films [16]. In addition, the tensile strength of the FK/PVA/Tris blend films are comparable to the FK/PVA blend film [18], and the toughness of the FK/PVA/Tris blend system is better.

Furthermore, for constant FK/PVA compositions (i.e., P-40), the Tris content influenced the tensile mechanical properties of the films. Upon increasing the Tris content from 15% to 30%, the elastic modulus decreased from 2007.31 to 311.33 MPa, the elongation at break increased from 1.5% to 23.67%, and the tensile strength reduced from 22.89 to 8.12 MPa. These observations can be attributed to hydrogen bonding between Tris and the hydrophilic groups present in the FK and PVA chains, in addition to breakage of hydrogen bonds between the protein chains and the PVA molecules. The free volume between these species also increased. As such, upon increasing the Tris content, the mobility of the blend film was enhanced and the elongation at break increased. A similar result was reported for a glycerol-plasticized FK membrane prepared by a hot-pressing method [1]. Upon increasing the Tris content further to 40%, the elongation at break decreased from 23.67% to 16.67%, the elastic modulus increased from 311.33 to 422.16 MPa, and the tensile strength decreased to 5.92 MPa. Therefore, at Tris contents exceeding 30%, the mechanical properties of the blend films deteriorated.

In addition, Table 2 shows that the elongation at break of G-40-0 is equivalent to P-40-15, and the tensile strength is equivalent to P-40-30. This result indicated the toughness of the FK/PVA/Tris blend membrane is better than G-40-0, and its tensile performance is better than G-40-0 (Tris content is less than 30%); comparing P-40 with G-40-15, the toughness of the FK/PVA/Tris blend membrane is not as good as the glycerol-plasticized FK/PVA, but the tensile performance of the FK/PVA/Tris blend membrane is better than the glycerol-plasticized FK/PVA.

### 3.5. Moisture Sensitivity of the Blend Films

To determine the moisture sensitivity of a material, the magnitude of the water contact angle (θ) on the film surface can be employed: a smaller contact angle indicates a more hydrophilic surface. More specifically, when θ > 90°, the surface is considered hydrophobic, and when θ < 90°, the surface is considered hydrophilic.

**Figure 4:**
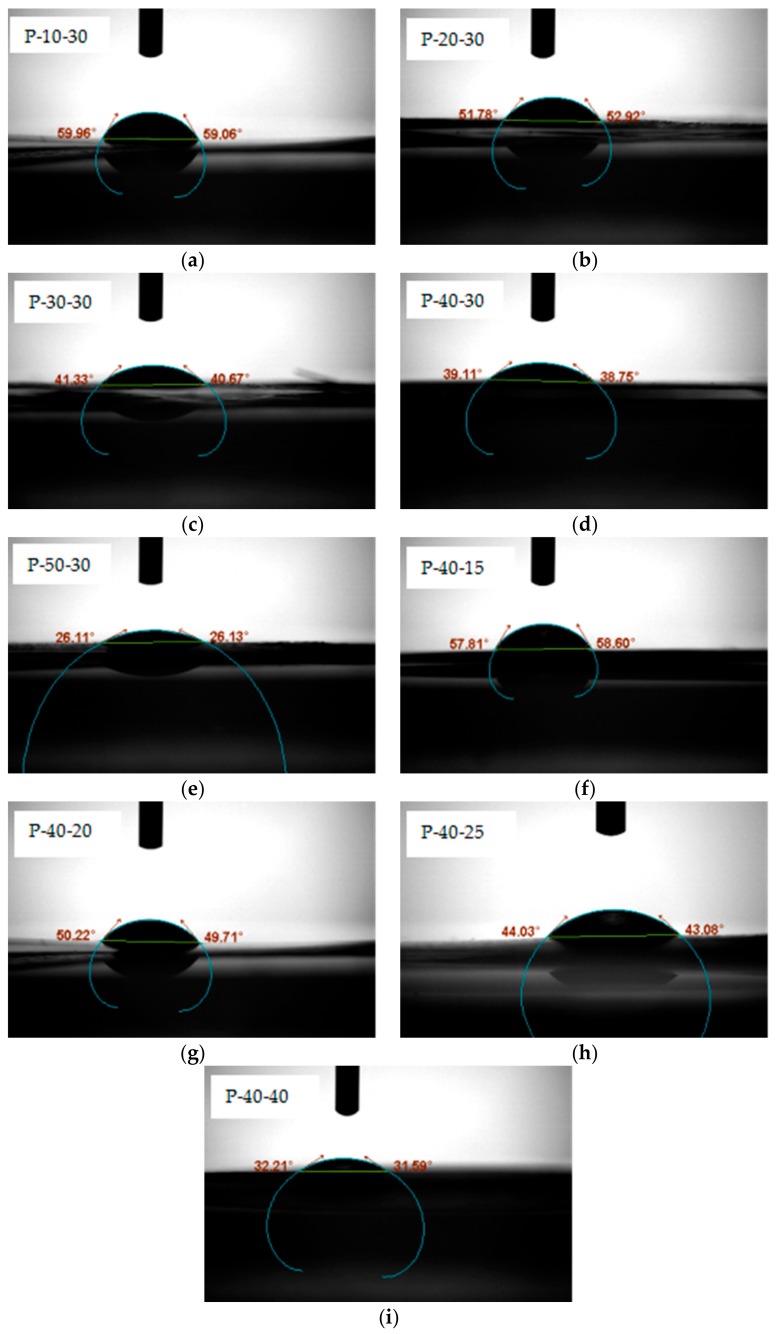
The contact angle of (**a**) P-10-30; (**b**) P-20-30; (**c**) P-30-30; (**d**) P-40-30; (**e**) P-50-30; (**f**) P-40-15; (**g**) P-40-20; (**h**) P-40-25; and (**i**) P-40-40.

To determine the effect of PVA content on film wettability, we investigated the contact angles of P-10, P-20, P-30, P-40, and P-50 containing 30 wt% Tris. For each sample, the contact angles were examined five seconds after the addition of a water droplet onto the film surface. As shown in the photographic images (Figure 4), the contact angles range from 0° to 90°, indicating that the FK/PVA/Tris blend films were wetted. In addition, at greater PVA contents, smaller contact angles were obtained; therefore, it was apparent that the moisture sensitivity increased in the presence of PVA, likely due to the large numbers of hydrophilic hydroxyl groups in the PVA structure. More specifically, the contact angles of P-10-30, P-20-30, P-30-30, P-40-30, and P-50-30 decreased as follows: 59.51°, 52.34°, 41°, 38.93°, and 26.12°.

Similarly, to determine the effect of the plasticizer on the film wettability, we investigated the contact angles of P-40 samples containing various Tris contents, as described above for variation in the PVA content. As shown in Figure 4, relatively high contact angles were observed for P-40-15, P-40-20, P-40-25, P-40-30, and P-40-40. However, the addition of Tris reduced the contact angles, indicating that the moisture sensitivity of these films increased in the presence of a plasticizer. A similar result was reported for glycerol-plasticized SPI/PVA films [16]. We therefore concluded that although Tris can improve the flexibility of FK/PVA films, it also enhances their moisture sensitivity.

### 3.6. Barrier Properties of the Prepared Films

The barrier properties of films are particularly important when considering their application as packaging materials. For example, high barrier packaging materials can effectively prevent the intrusion of small gas molecules (e.g., oxygen and water vapor) and microorganisms, in addition to preventing the escape of aromas. As such, the use of high barrier packaging materials can ensure the stability of the internal package environment and prolong the shelf life of the product [30]. The suitability for blend films for application in the packaging field is determined by their barrier properties. In this case, we selected the water vapor permeability and the oxygen permeability of the blend films for examination.

#### 3.6.1. Water Vapor Permeability (WVP)

The WVP of a material is typically affected by its hydrophilicity, flaws, and the internal tortuosity of the structure. Thus, the effects of the PVA and Tris contents on the WVP of the blend films were examined and the results are outlined in Table 3. More specifically, the WVP value of the Tris-plasticized FK/PVA films ranged from 1.47 × 10^−12^ to 3.68 × 10^−12^ g·cm^−1^·s^−1^·Pa^−1^, lower than the corresponding values reported for glycerol-plasticized SPI/PVA films prepared by hot-pressing (i.e., 2.03 × 10^−11^ to 3.75 × 10^−11^ g·cm^−1^·s^−1^·Pa^−1^) [15], but higher than the corresponding values reported for dialdehyde starch-crosslinked FK/PVA films prepared by solution-casting (i.e., 5 × 10^−13^ to 1.32 × 10^−12^ g·cm^−1^·s^−1^·Pa^−1^) [18]. As indicated in the table, the WVP values of the FK/PVA/Tris films increased with increasing PVA content, likely due to the hydrophilic nature of PVA caused by large numbers of hydroxyl groups in its chain. Thus, upon increasing the PVA content, the film hydrophilicity increased, as confirmed by contact angle measurements. Notably, the WVP values of the FK/PVA/Tris films also increased with increasing Tris content, which is common in protein-based films plasticized with glycerol. In this case, as the free volume of the system is increased due to the plasticization effect, the protein network becomes sparse, thereby increasing the number of pathways available for the transfer of water molecules within the films. As such, the water vapor barrier properties of the blend films are lowered upon increasing both the PVA and the Tris contents.

In addition, comparing the WVP value of FK/PVA/Tris blend films (P-40) with G-40-0 and G-40-15, it was found that the WVP value of G-40-0 was lower than the FK/PVA/Tris blend films (except P-40-15) and the WVP value of G-40-15 was higher than the FK/PVA/Tris blend films. That is to say, the water vapor barrier performance of the FK/PVA/Tris blend system is worse than that of the FK/PVA blend system without Tris, but better than glycerol-plasticized FK/PVA.

#### 3.6.2. Oxygen Permeability (OP)

The effects of the PVA and Tris contents on the OP values of the Tris-plasticized FK/PVA films are outlined in Table 3. More specifically, the OP values ranged from 3.214 × 10^−5^ to 5786 × 10^−5^ cm^3^·m^−2^·d^−1^·Pa^−1^. Due to the poor mechanical properties of P-10-30, P-20-30, P-30-30, and G-40-0, cracks were generated on the blend film surfaces under the test conditions; for the control film G-40-15, due to the plasticization effect of glycerin, the free volume in the film is increased and O_2_ is easily transmitted through G-40-15. Therefore, the OP values of the films were too large to be measured, as they were out of the instrument range. In general, the oxygen barrier properties of FK/PVA/Tris blend films are superior to the control films. In addition, we found that the OP values of the composite films decreased with increasing PVA and Tris content, a trend opposite to the one observed for the WVP. It should be noted here that the process of air permeation in a film generally involves three stages: adsorption, diffusion, and desorption. Since the hydrophilicity of the FK/PVA/Tris blend film surfaces increased with increasing PVA and Tris content, the adsorption of non-polar O_2_ on the film surface is inhibited. As such, the oxygen barrier properties of the blend films were enhanced upon increasing both the PVA and Tris content.

## 4. Conclusions

We herein reported the preparation of blend films from feather keratin (FK), synthetic poly(vinyl alcohol) (PVA), and tris(hydroxymethyl)aminomethane (Tris) using a solution-casting process. A phase separation occurred in the FK/PVA/Tris blended system, which was confirmed by scanning electron microscopy. The strong hydrogen bonding interactions present between FK, PVA and Tris were confirmed by Fourier transform infrared spectroscopy and powder X-ray diffraction analysis. We found that upon increasing the quantity of PVA present in the blend, the elastic modulus and water vapor barrier property of the films were reduced, while the elongation at break, hydrophilicity, and oxygen barrier property were enhanced. These variations were attributed to the strong hydrophilicity and excellent mechanical properties of PVA. In addition, an increase in the Tris content increased the hydrophilicity, water vapor permeability, and elongation at break. We therefore concluded that Tris plays the role of a plasticizer in the blend film. Moreover, the method reported herein allows the preparation of tuneable Tris-plasticized FK/PVA blend films from cheap, biodegradable, and ecofriendly materials, thereby constituting a convenient and promising means to prepare FK plastics for practical packaging applications.

## 5. Patents

The research results have been patented and the application number is CN201810587364.2.

## Figures and Tables

**Table 1:** Effect of the blend film PVA content on the FK secondary structure.

Sample	α-Helix (%)	β-Turn (%)	β-Sheet (%)	Random Coil (%)
P-0-30	13.98	24.3	39.55	19.89
P-10-30	13.8	23.87	40.43	19.89
P-20-30	14.1	23.63	39.77	20.3
P-30-30	14.89	26.36	36.53	20.49
P-40-30	14.78	26.8	36.14	20.65
P-50-30	15.66	26.29	35.62	21.51

The secondary structures were expressed as a percentage of corresponding area by ratio to the total amide I band area.

**Table 2:** Tensile properties of the prepared films.

Sample	Elastic Modulus (MPa)	Elongation at Break (%)	Tensile Strength (MPa)	Thickness (mm)
P-10-30	493.66 ± 21.98	1.75 ± 0.35	7.03 ± 0.68	0.1 ± 0.005
P-20-30	324.41 ± 16.75	2 ± 0	5.07 ± 0.1	0.117 ± 0.004
P-30-30	327.97 ± 38.95	7 ± 0.71	8.98 ± 0.07	0.094 ± 0.002
P-40-30	311.33 ± 25.05	23.67 ± 3.53	8.12 ± 0.59	0.081 ± 0.003
P-50-30	214.55 ± 16.13	179.83 ± 9.11	9.52 ± 1.07	0.08 ± 0.004
P-40-15	2007.31 ± 38.76	1.5 ± 0	22.89 ± 0.22	0.067 ± 0.002
P-40-20	742.74 ± 45.86	2.67 ± 0.16	12.2 ± 0.87	0.076 ± 0.008
P-40-25	416.78 ± 17.34	10.83 ± 1.01	9.58 ± 0.37	0.087 ± 0.004
P-40-30	311.33 ± 25.05	23.67 ± 3.53	8.12 ± 0.59	0.081 ± 0.004
P-40-40	422.16 ± 45.12	16.67 ± 0.91	5.92 ± 0.2	0.103 ± 0.003
G-40-0	1257.18 ± 28.58	1.5 ± 0	8.95 ± 0.01	0.081 ± 0
G-40-15	15.81 ± 5.19	262 ± 0.71	3.87 ± 0.27	0.081 ± 0.006

**Table 3:** Water vapor permeability (WVP) and oxygen permeability (OP) values for the prepared films.

Sample	WVP (×10^−12^ g·cm^−1^·s^−1^·Pa^−1^)	OP (×10^−5^ cm^3^·m^−2^·d^−1^·Pa^−1^)
P-10-30	–	–
P-20-30	2.74 ± 0.16	–
P-30-30	3 ± 0.26	–
P-40-30	3.36 ± 0.18	7.907 ± 0.35
P-50-30	3.68 ± 0.1	3.214 ± 0.26
P-40-15	1.47 ± 0.08	5786 ± 6.7
P-40-20	1.87 ± 0.11	28.72 ± 1.2
P-40-25	3.09 ± 0.1	11.78 ± 0.65
P-40-30	3.36 ± 0.18	7.907 ± 0.35
P-40-40	2.66 ± 0.09	13.78 ± 1.08
G-40-0	1.7 ± 0.05	–
G-40-15	3.43 ± 0.14	–
